# The heat shock response plays an important role in TDP-43 clearance: evidence for dysfunction in amyotrophic lateral sclerosis

**DOI:** 10.1093/brain/aww028

**Published:** 2016-03-01

**Authors:** Han-Jou Chen, Jacqueline C. Mitchell, Sergey Novoselov, Jack Miller, Agnes L. Nishimura, Emma L. Scotter, Caroline A. Vance, Michael E. Cheetham, Christopher E. Shaw

**Affiliations:** ^1^ Maurice Wohl Clinical Neuroscience Institute, Institute of Psychiatry, Psychology and Neuroscience, King’s College London, London, UK; ^2^ UCL Institute of Ophthalmology, 11-43 Bath Street, London, UK; ^3^ Department of Pharmacology, University of Auckland, New Zealand

**Keywords:** TDP-43 proteinopathy, ALS, heat shock response, HSF1, molecular chaperone

## Abstract

Detergent-resistant, ubiquitinated and hyperphosphorylated Tar DNA binding protein 43 (TDP-43, encoded by *TARDBP* ) neuronal cytoplasmic inclusions are the pathological hallmark in ∼95% of amyotrophic lateral sclerosis and ∼60% of frontotemporal lobar degeneration cases. We sought to explore the role for the heat shock response in the clearance of insoluble TDP-43 in a cellular model of disease and to validate our findings in transgenic mice and human amyotrophic lateral sclerosis tissues. The heat shock response is a stress-responsive protective mechanism regulated by the transcription factor heat shock factor 1 (HSF1), which increases the expression of chaperones that refold damaged misfolded proteins or facilitate their degradation. Here we show that manipulation of the heat shock response by expression of dominant active HSF1 results in a dramatic reduction of insoluble and hyperphosphorylated TDP-43 that enhances cell survival, whereas expression of dominant negative HSF1 leads to enhanced TDP-43 aggregation and hyperphosphorylation. To determine which chaperones were mediating TDP-43 clearance we over-expressed a range of heat shock proteins (HSPs) and identified DNAJB2a (encoded by *DNAJB2* , and also known as HSJ1a) as a potent anti-aggregation chaperone for TDP-43. DNAJB2a has a J domain, allowing it to interact with HSP70, and ubiquitin interacting motifs, which enable it to engage the degradation of its client proteins. Using functionally deleted DNAJB2a constructs we demonstrated that TDP-43 clearance was J domain-dependent and was not affected by ubiquitin interacting motif deletion or proteasome inhibition. This indicates that TDP-43 is maintained in a soluble state by DNAJB2a, leaving the total levels of TDP-43 unchanged. Additionally, we have demonstrated that the levels of HSF1 and heat shock proteins are significantly reduced in affected neuronal tissues from a TDP-43 transgenic mouse model of amyotrophic lateral sclerosis and patients with sporadic amyotrophic lateral sclerosis. This implies that the HSF1-mediated DNAJB2a/HSP70 heat shock response pathway is compromised in amyotrophic lateral sclerosis. Defective refolding of TDP-43 is predicted to aggravate the TDP-43 proteinopathy. The finding that the pathological accumulation of insoluble TDP-43 can be reduced by the activation of HSF1/HSP pathways presents an exciting opportunity for the development of novel therapeutics.

## Teaser

Insoluble TDP-43 inclusions are the pathological hallmark of ALS and tau-negative frontotemporal lobar degeneration. Chen *et al.* show that the heat shock response (HSR), which regulates chaperone expression, is compromised in an ALS mouse model and in patients. Activation of the HSR clears insoluble TDP-43 and increases cell survival.

## Graphical abstract

**Figure:**
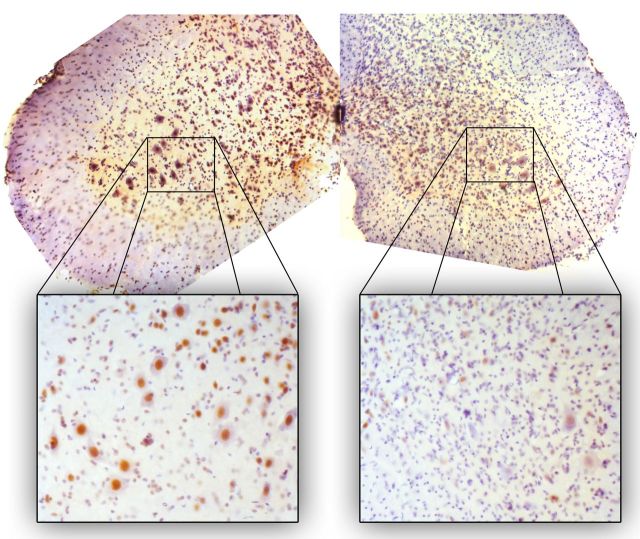
Insoluble TDP-43 inclusions are the pathological hallmark of ALS and tau-negative frontotemporal lobar degeneration. Chen *et al.* show that the heat shock response (HSR), which regulates chaperone expression, is compromised in an ALS mouse model and in patients. Activation of the HSR clears insoluble TDP-43 and increases cell survival.

## Introduction

Amyotrophic lateral sclerosis (ALS) is the most common adult-onset motor neuron degenerative disease, characterized by progressive motor neuron loss from the spinal cord, brainstem and motor cortex, leading to muscle weakness and eventual respiratory failure. The average age of disease onset is 55 years and affected individuals typically die within 3 to 5 years of diagnosis. No effective treatments are available and consequently there is a high unmet need for novel therapeutic development (for review see Goyal and Mozaffar, 2014 ). Approximately 10% of ALS cases are familial, with the remaining 90% being sporadic (sporadic ALS). Regardless of the cause, ∼95% of patients share a common molecular pathology, which involves the accumulation of ubiquitinated, hyperphosphorylated and insoluble TDP-43 protein aggregates in the cytoplasm, and decreased levels of TDP-43 in the nucleus ( Neumann *et al.* , 2006 ; Mackenzie *et al.* , 2007 ; Tan *et al.* , 2007 ; Geser *et al.* , 2008 ; Brettschneider *et al.* , 2013 ).

TDP-43 is a DNA and RNA binding protein that plays a key role in regulating RNA transcription, editing, transport and translation. It is a predominantly nuclear protein whose levels are tightly controlled through a system of auto-regulation ( Ayala *et al.* , 2011 ; Polymenidou *et al.* , 2011 ). Knockout of TDP-43 is lethal in embryonic mice, indicating that it plays a vital role in development and survival ( Kraemer *et al.* , 2010 ). Although TDP-43 is predominantly a nuclear protein, it is known to shuttle between the nucleus and cytoplasm and has been identified as a component of stress granules, which form in response to cellular stress ( Belly *et al.* , 2005 ; Rutherford *et al.* , 2008 ; Colombrita *et al.* , 2009 ; Dion *et al.* , 2009 ; Nonaka *et al.* , 2009 ). Multiple ALS-causing mutations have been identified on the *TARDBP* gene coding for TDP-43, which promotes TDP-43 translocation to the cytosol, stabilizes and promotes the aggregation of the mutant protein ( Johnson *et al.* , 2009 ; Barmada *et al.* , 2010 ; Ling *et al.* , 2010 ). TDP-43 is aggregation-prone and the accumulation of mutant protein in the cytoplasm correlates with toxicity ( Barmada *et al.* , 2010 ; Bilican *et al.* , 2012 ). In addition, overexpression of wild-type TDP-43 in mammalian cells or transgenic animal models leads to TDP-43 accumulation, cytotoxicity and motor deficits ( Ash *et al.* , 2010 ; Barmada *et al.* , 2010 ; Stallings *et al.* , 2010 ; Wils *et al.* , 2010 ). As TDP-43 cytoplasmic aggregates are observed in 95% of ALS and tau-negative frontotemporal lobar degeneration cases, dysregulation of TDP-43 protein levels are a common feature of disease pathogenesis. The targeted clearance of aggregated TDP-43 is therefore a key strategy for therapeutic intervention.

A balance of protein synthesis, folding and degradation is needed to maintain protein homeostasis (proteostasis) and this process is facilitated by molecular chaperones. When cells are acutely stressed by insults that affect proteostasis, such as heat shock, they induce a transcriptional programme orchestrated by the transcription factor heat shock factor 1 (HSF1) to upregulate selected chaperones, known as heat shock proteins (HSPs), to protect them from being overwhelmed by the accumulation of aberrant proteins. HSPs are categorized into several families according to their function and size (for review see Stetler *et al.* , 2010 ). HSP90 and HSP70 are families of classic abundant chaperones that are expressed ubiquitously in most subcellular organelles. They execute their chaperone function by facilitating the folding of client proteins in an ATP-dependent manner. The human DnaJ (HSP40) family contains ∼50 proteins that act as co-chaperones for HSP70 to promote the client–chaperone interaction by stimulating HSP70 ATP hydrolysis, producing a high affinity binding state for client protein refolding. Some members of the HSP40 family have HSP70-independent functions and can facilitate client proteins folding on their own or directly targeting them for degradation ( Kampinga and Craig, 2010 ).

Aberrant protein aggregation is a pathological hallmark of almost all neurodegenerative diseases and the beneficial effect of increasing individual HSP expression has been demonstrated for several neurodegenerative disease models. For example, overexpression of the co-chaperones, DNAJB6b and DNAJB8, suppressed the aggregation and toxicity of poly-Q proteins ( Hageman *et al.* , 2010 ); overexpression of the small HSP, HSPB8, promoted autophagic removal of mutant SOD1 ( Crippa *et al.* , 2010 ); overexpression of HSP70 inhibited α-synuclein fibril formation ( Dedmon *et al.* , 2005 ); overexpression of HSPB1 in mutant SOD1 transgenic mice improved neuronal survival in the early but not late stages of disease development ( Sharp *et al.* , 2008 ); and overexpression of DNAJB2a in SOD1 transgenic mice reduced SOD1 aggregation ( Novoselov *et al.* , 2013 ).

In this study, we explored the potential of HSPs to clear pathological TDP-43 aggregates and whether these chaperones might be deficient in animal models of TDP-43 proteinopathy and patients with ALS. We established a cellular model that recapitulated key features of the human TDP-43 proteinopathies and demonstrated that overexpression of dominant active HSF1, or the co-chaperone DNAJB2a, dramatically cleared insoluble TDP-43 aggregates and improved cell survival without altering levels of soluble TDP-43. We demonstrated that DNAJB2a mediates pathological TDP-43 clearance through a ubiquitin proteasome system (UPS)-independent, HSP70-dependent mechanism. This suggests that the HSF1-DNAJB2a pathway refolds TDP-43 and returns it to its natural physiological state rather than facilitating its degradation. We also demonstrated that the levels of HSF1 and HSPs are markedly reduced in both a transgenic TDP-43 mouse model and in spinal cord tissues from sporadic ALS patients. These results highlight the potential of HSF1 and HSP activation as an important therapeutic strategy for the TDP-43 proteinopathies.

## Materials and methods

### Plasmids and antibodies

**Figure 1:**
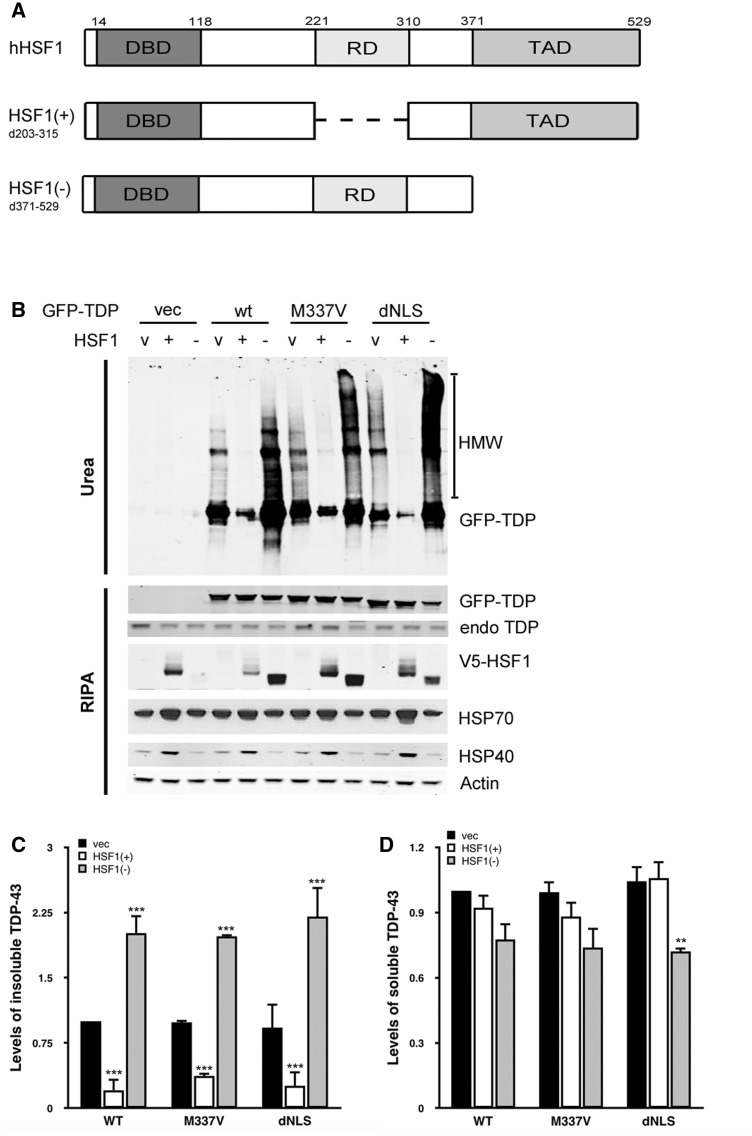
**Expression of HSF1(+) reduces levels of insoluble TDP-43.** ( **A** ) Protein structure of human HSF1 protein and the HSF1 variants used in this study. ( **B** ) HEK293T cells co-express GFP-TDP-43 and V5-HSF1 constructs for 48 h followed by fractionation. Levels of endogenous HSP40 and HSP70 are shown as indicators of HSR activation. Levels of GFP-TDP-43 from three independent transfections are quantified, normalized to actin and shown in relation to GFP-WT-TDP-43 and the vector only control. Mean and SEM are shown for insoluble fraction ( **C** ), and soluble fraction ( **D** ) **** . Two-way ANOVA analysis indicates the difference in TDP-43 solubility is not contributed by TDP-43 genotypes or interaction between TDP-43 and HSF1, but by HSF1 alone ( *P <* 0.001). Both HSF1(+) and HSF1(−) cause significant changes in the level of insoluble TDP-43 when co-transfected with GFP-TDP-43 when compared to the vector only control ( *P <* 0.001, Bonferroni post-test).

The green fluorescent protein (GFP)-TDP-43 constructs were generated and used in a previous study ( Nishimura *et al.* , 2010 ). HSF1(+) and HSF1(−) ( Fig. 1 A) were generated by altering the HSF1 constructs kindly provided by Professor Richard Voellmy ( Xia *et al.* , 1999 ). They were cloned with a V5 tag fused to the N-terminus into the pcDNA5/FRT/TO expression vector (Life Technologies). V5-chaperone constructs were kindly provided by Professor Harm Kampinga ( Hageman and Kampinga, 2009 ) and Myc-DNAJB2a constructs were generated and used in previous studies ( Chapple and Cheetham, 2003 ; Westhoff *et al.* , 2005 ). All plasmid sequences were verified by DNA sequencing.

Primary antibodies used in this study included: rabbit anti-ubiquitin, K48-specific (1:2000, Millipore); mouse anti-phospho TDP-43 (1:3000, Cosmo Bio); rabbit anti-mouse TDP-43 (0.1 μg/ml, a gift from Professor Virginia Lee; Igaz *et al.* , 2011 ); mouse anti-Myc (9B11; 1:1000 for immunoblotting, 1:100 for immunopurification, Cell Signaling); rabbit anti-HSP40 (C64B4; 1:1000, Cell Signaling); rabbit anti-HSP70 (1:1000, Cell Signaling); rabbit anti-HSF1 (1:1000, Cell Signaling); mouse anti-Actin (1:4000, Sigma); rabbit anti-V5 (1:2000 for immunoblotting, 1:1000 for immunopurification, Sigma); mouse anti-GFP (1:1000, Santa Cruz); rabbit anti-TDP-43 (1:2000, ProteinTech); and rabbit anti-p62 (1:10 000, Abcam). Secondary antibodies included DyLight® 680 goat anti-rabbit IgG (1:10 000, Thermo Scientific); DyLight® 800 goat anti-mouse IgG (1:10 000, Thermo Scientific); HRP-linked anti-rabbit IgG (1:2000, Cell Signaling); and DyLight® 488/550/650 anti-rabbit or mouse IgG (1:500, Thermo Scientific).

### Cell culture and DNA transfection

HEK293T and SH-SY5Y cells were cultured using Dulbecco’s modified Eagle medium (DMEM) and DMEM/F12 (Life Technologies) supplemented with 10% foetal bovine serum (Life Technologies), and maintained at 37°C, 5% CO _2_ . Cells were plated a day before transfection and media were refreshed before plasmid DNA transfection using Lipofectamine® 2000 (Life Technologies). Cells were left for 48 h after transfection to be harvested for analysis unless otherwise stated.

### Animals

All experiments were performed under the terms of the UK Animals (Scientific Procedures) Act 1986, and were approved by the Kings College, London ethics review panel. Compound transgenic mice were generated by crossing TDP-43 ^WT^ and TDP-43 ^Q331K^ mice as previously described ( Mitchell *et al.* , 2015 ).

### Mouse and human spinal cord lysate preparation

Whole spinal cords from 8-week-old TDP-43 ^WTxQ331K^ animals and their single and non-transgenic littermates ( *n =* 3–4) or 50 mg of spinal cord post-mortem tissues from controls or patients with sporadic ALS were homogenized in the appropriate amount of RIPA buffer (50 mM Tris pH8.0, 150 mM NaCl, 1% NP-40, 0.5% sodium deoxycholate, 0.1% sodium dodecyl sulphate with protease and phosphatase inhibitor), sonicated and stored at −80 °C. Protein concentration was determined with DC™ Protein Assay Kit (Bio-Rad). Western blotting was run with 10 μg of protein lysate.

### Immunohistochemistry

Eight-week-old, end-stage TDP-43 ^WTxQ331K^ mice and age-matched littermates were anaesthetized and transcardially perfused with phosphate-buffered saline (PBS) followed by 4% paraformaldehyde (PFA) in phosphate buffer. Spinal cord was postfixed in 4% PFA in 15% sucrose for 5 h, cryoprotected in 30% sucrose for 24 h and cut into 30 µm sections on a cryostat. Sections were incubated in rabbit anti-HSF1 antibody (1:250; Cell Signaling), washed and incubated with a biotinylated anti-rabbit antibody (1:1000; Vector) and then incubated in an ABC kit (Vector). Sections were imaged using a Zeiss light microscope and Axiovision software.

### Immunopurification

Cells were harvested in immunopurification buffer (50 mM Tris pH7.4, 150 mM NaCl, 1% Triton™ X-100 with protease and phosphatase inhibitor). After centrifuging (14 000 rpm for 30 s at 4°C), the supernatant was collected and incubated with immunopurification antibody and Dynabead protein A (Life Technologies) overnight at 4°C. The Dynabead® protein A-antibody-protein complex was purified using magnetic separation and washed with immunopurification buffer before eluted in loading buffer.

### Solubility fractionation

The fractionation for protein solubility was performed using a protocol described by Winton *et al.* (2008) with some minor modifications. Cells were harvested in RIPA buffer, sonicated and centrifuged at 12 000 *g* for 20 min at 4 °C. After centrifugation, the supernatant was collected as the RIPA solubility fraction. The pellet, after being washed once with RIPA buffer, was then suspended in 20% of the original lysis volume with urea buffer (7 M Urea, 2 M Thiourea, 4% CHAPS and 30 mM Tris pH8.5) and collected as the insoluble, detergent-resistant fraction.

### Western blotting and densitometry analysis

Protein quantification and western blotting were performed as described before ( Nishimura *et al.* , 2010 ). Five micrograms of cell lysate from the RIPA fraction and the equivalent liquid volume from the urea fraction were loaded. Western blot quantification was performed using the image analysis software, ImageJ ( http://imagej.nih.gov/ij/ ). Integrated band intensities were normalized to that of loading control or the RIPA fraction.

### Immunofluorescence

Cells for immunofluorescent analyses were fixed in 4% PFA (VWR) for 20 min and washed with PBS three times for 5 min. Cells were permeablized by incubation in PBS containing 0.5% Triton™ X-100 (Sigma) for 15 min at room temperature, followed by blocking in PBS containing 1% donkey serum for 1 h at room temperature. Cells were incubated with primary antibody diluted in blocking solution overnight at 4°C. After washing in PBS, cells were subsequently incubated with fluorescent secondary antibodies diluted in blocking solution for 1 h at room temperature. DAPI (Sigma) was then used to stain for nuclei before being mounted on coverslips using FluroSave (Calbiochem).

### Cell survival assay

For cell survival assay, SH-SY5Y cells were trypsinized and stained with Calcein Violet 450 AM Viability Dye (500 nM, eBioscience) for 30 min at room temperature. After washing twice with PBS, cells were resuspended in PBS and analysed with the BD FACSCanto II (BD Biosciences).

## Results

### Co-expression of HSF1(+) reduces levels of insoluble TDP-43 protein

This study used dominant active and negative forms of HSF1 to investigate the effect of the HSR and HSP upregulation on TDP-43 proteinopathy in cellular models. HSF1 is a transcription factor that upregulates HSPs during the stress response. In the unstressed condition, HSF1 is bound to HSP90 via its regulatory domain and is kept in an inactive form. During cellular stress, HSF1 dissociates from HSP90, trimerizes, translocates into the nucleus, and binds to the heat shock element (HSE) through its DNA binding domain (DBD), where it promotes the transcription of downstream genes via its transactive domain (TAD) ( Voellmy, 2005 ; Velichko *et al.* , 2013 ). To activate the HSR we used a dominant positive HSF1, HSF1(+), in which the regulatory domain is deleted ( Fig. 1 A). This modified HSF1 is therefore unable to be inactivated by HSP90 binding. Conversely, for comparison, we used a dominant negative construct, HSF1(−), with the TAD domain deleted ( Fig. 1 A). Although this HSF1(−) can still form trimers with endogenous wild-type HSF1 during stress, it is incapable of inducing the transcription of its targeted genes. The expression of HSF1(+) increased the levels of endogenous HSP40 (DNAJB1) and HSP70 (HSPA1A), demonstrating HSF1(+) activated the HSR as expected ( Fig. 1 B). On the other hand, levels of HSP40 and HSP70 were unchanged, or slightly decreased in HSF1(−) expressing cells, indicating HSF1(−) overexpression did not induce the HSR, and furthermore, may even inhibit basal HSR activation by dominant negative inhibition of endogenous HSF1.

The expression level of endogenous TDP-43 in our cells was not high enough to cause any proteinopathy phenotype similar to that seen in disease tissues, and was not found in the insoluble fraction ( Supplementary Fig. 1 A and B). Overexpression of wild-type or mutant TDP-43 in HEK293 cells resulted in high levels of TDP-43 accumulating in the detergent-resistant fraction ( Fig. 1 B and C). This result is consistent with previous reports showing that high cellular levels of TDP-43 caused protein aggregation resulting in a decrease in protein solubility. However, co-expression of TDP-43 with HSF1(+) led to a significant reduction in the level of insoluble TDP-43 to ∼25% ( Fig. 1 B and C, *P <* 0.001). Conversely, HSF1(−) significantly increased the level of insoluble TDP-43 by ∼250% ( Fig. 1 B and C, *P <* 0.001). The mutation status of TDP-43 when co-transfected with HSF1 did not significantly change TDP-43 protein solubility, nor did the presence of a mutation in TDP-43 when transfected alone ( Fig. 1 C). HSF1(+) showed no obvious effect on the level of soluble TDP-43 ( Fig. 1 B and D). However, soluble GFP-TDP-43 was found to slightly decrease when co-expressed with HSF1(−) especially for the cytosolic TDP-43, GFP-dNLS TDP-43 ( Fig. 1 B and D, *P <* 0.01 for dNLS-TDP-43), suggesting the expression of HSF1(−) results in an increased tendency for overexpressed cytosolic TDP-43 to shift from soluble monomer to insoluble aggregates.

In summary, these results show that increased activation of the HSR by HSF1(+) substantially enhances the clearance of insoluble TDP-43, whereas inhibition of the HSR via HSF1(−) expression results in an increase in insoluble TDP-43, likely resulting from an inhibition of the endogenous, basal HSR.

### HSF(+) rescues TDP-43 hyperphosphorylation

**Figure 2:**
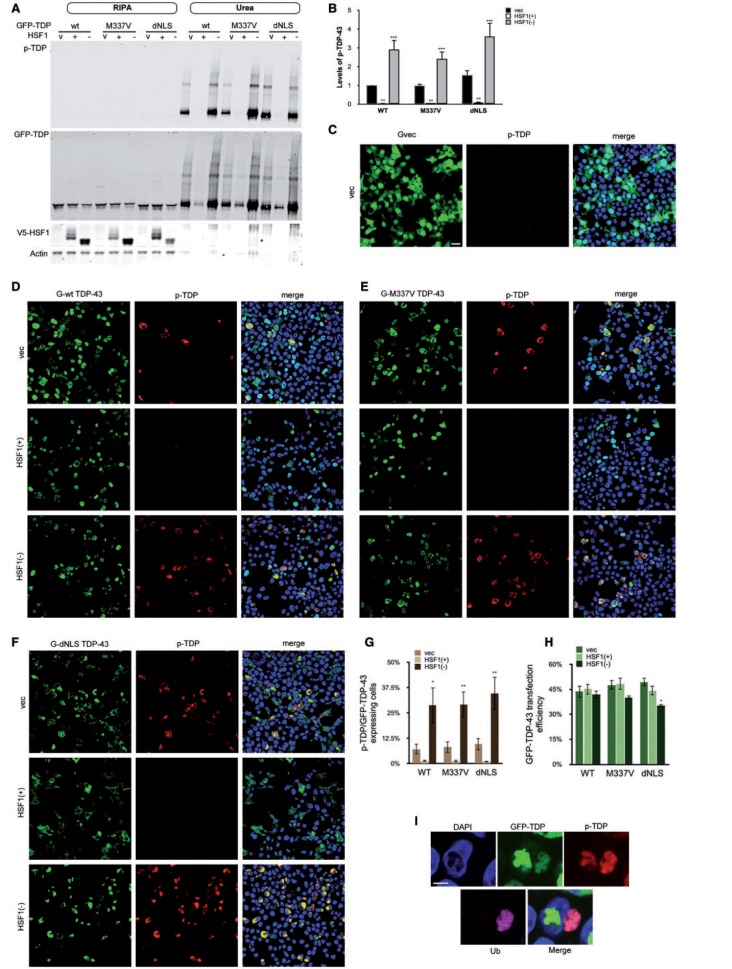
**Expression of HSF1(+)rescues TDP-43 phosphorylation.** ( **A** ) HEK293T cells co-expressing GFP-TDP-43 and V5-HSF1 constructs for 48 h followed by fractionation. Levels of phosphorylated TDP-43 from three independent transfections were quantified, normalized to actin, and shown in relation to the GFP-wild-type TDP-43 and the vector only control. Mean and SEM are shown in ( **B** ). Two-way ANOVA analysis indicates that the difference in TDP-43 phosphorylation is not contributed by TDP-43 genotypes or interaction between TDP-43 and HSF1, but by HSF1 alone ( *P <* 0.001). When co-transfected with GFP-TDP-43, both HSF1(+) and HSF1(−) significantly changed the level of phosphorylated TDP-43 compared to the vector only control ( *P =* 0.02 and *P <* 0.001, respectively, Bonferroni post-test). ( **C** – **F** ) Phospho-TDP-43 staining in HEK293T cells transfected for 48 h with the V5-HSF1 and GFP-TDP-43 vectors ( **C** ) **,** GFP-WT-TDP-43 ( **D** ), GFP-M337V TDP-43 ( **E** ) and GFP-dNLS TDP-43 ( **F** ). Scale bar = 20 μm in **C** . Cell counting was performed on three independent transfections, with 300 cells per condition. Mean and SEM of frequency of GFP-TDP-43 expressing cells that are also phospho-TDP-43 stained positive ( **G** ) and GFP-TDP-43 transfection efficiency ( **H** ) are shown. ( **I** ) GFP-WT-TDP-43 and HSF1(−) transfected HEK293T cell stained for phospho-TDP-43 (red) and ubiquitin (magenta). Scale bar = 5 μm.

Aside from reduced solubility, TDP-43-linked pathology is also characterized by the presence of hyperphosphorylated TDP-43, which can be detected by a phospho-TDP-43 specific antibody. Phospho-TDP-43 was only detectable in the insoluble urea fraction, confirming that the pathological TDP-43 in our model is both insoluble and hyperphosphorylated ( Fig. 2 A). The accumulation of insoluble, phosphorylated TDP-43 was completely inhibited in HSF1(+) co-expressing cells ( Fig. 2 A and B, *P <* 0.01) and enhanced by HSF1(−) ( Fig. 2 A and B, *P <* 0.001). No phospho-TDP-43 staining was identified in GFP-vector transfected cells alone ( Fig. 2 C), indicating that TDP-43 phosphorylation was not caused by the stress of transient transfection but by the accumulation of aggregated TDP-43, which contained endogenous TDP-43 ( Supplementary Fig. 1 C). Staining for phospho-TDP-43 in cells confirmed the western blot findings, with evidence of TDP-43 phosphorylation in ∼10% of GFP-TDP-43 expressing cells. HSF1(+) substantially reduced levels of TDP-43 phosphorylation in both wild-type and mutant TDP-43 expressing cells without altering the TDP-43 transfection efficiency ( Fig. 2 D–H). On the other hand, HSF1(−) increased TDP-43 phosphorylation, which coincided with a marginal reduction in the frequency of GFP-dNLS TDP-43 expressing cells ( Fig. 2 H, *P =* 0.012). The genotype of the overexpressed TDP-43 had no significant impact on TDP-43 phosphorylation *per se* ( Fig. 2 B and G). We also observed a slight change in TDP-43 protein subcellular localization in this model, as cells that co-expressed GFP-TDP-43 and HSF1(+) tended to have a higher proportion of nuclear TDP-43, while cells that co-expressed GFP-TDP-43 and HSF1(−) displayed a higher proportion of cytosolic TDP-43 ( Supplementary Fig. 2 ). In the aggravated condition where TDP-43 was co-expressed with HSF1(−), not only was phosphorylated TDP-43 identified in both the nucleus and cytoplasm, but ∼30% of the cytosolic phosphorylated TDP-43 inclusions were also ubiquitin-positive ( Fig. 2 I).

**Figure 3:**
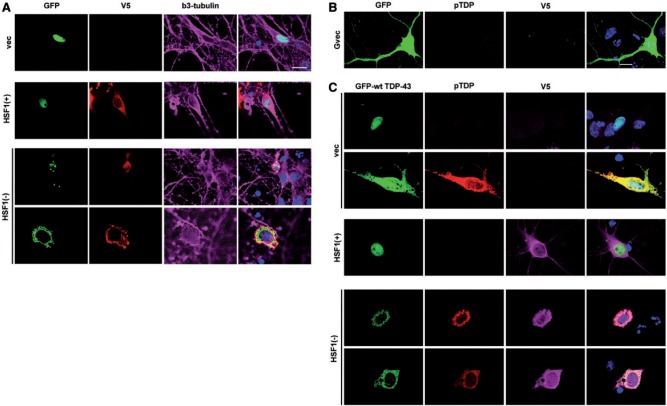
**Effect of HSF1 on TDP-43 cellular distribution and phosphorylation in primary neurons.** ( **A** ) Rat primary cortical neurons were transfected with GFP-WT-TDP-43 (green) and V5-HSF1 (red). βIII-tubulin (magenta) is used as a neuronal marker. ( **B** and **C** ) GFP vector ( **B** ) or GFP-wild-type TDP-43 with V5-HSF1 ( **C** ) are transfected into the rat primary cortical neurons. Cells were fixed 6 days after transfection and stained for V5 (magenta), phospho-TDP-43 (red) and nuclei (DAPI, blue). Scale bar = 10 μm.

To further validate these findings in a neuronal model, we overexpressed GFP-TDP-43 with HSF1 in rat primary cortical neurons ( Fig. 3 ). Overexpression of GFP vector did not induce TDP-43 phosphorylation in transfected neurons ( Fig. 3 B), which again demonstrates that exogenous protein expression is not the cause of TDP-43 hyperphosphorylation. When primary neurons were transfected with GFP-TDP-43 alone, expression was largely confined to nuclei (65.9 ± 1.48%) but cytoplasmic localization of phosphorylated TDP-43 was also observed in a significant minority of cells (31.2 ± 0.54%) ( Fig. 3 C, top). In HSF1(+)/GFP-TDP-43 expressing cells, all TDP-43 displayed a nuclear localization with no evidence of phosphorylation ( Fig. 3 C, middle). However, in cells co-expressing HSF1(−) and GFP-TDP-43, there was a dramatic reduction of nuclear TDP-43 and accumulation of hyperphosphorylated TDP-43 in the cytoplasm ( Fig. 3 C, bottom). The clearance of nuclear TDP-43 in cells containing large cytoplasmic aggregates is in agreement with observations of pathological TDP-43 in ALS patient tissues ( Neumann *et al.* , 2006 ). Of note, GFP-positive cells co-expressing HSF1(−) were very rare, and many had lost their neuron-like morphology ( Fig. 3 A and C), suggesting that TDP-43 cytotoxicity is enhanced when the HSR is suppressed.

Our cellular model of TDP-43 proteinopathy replicates several features of human disease with the accumulation of insoluble phosphorylated and ubiquitinated TDP-43 in the cytoplasm. We have also shown that the activation of the HSR rescues TDP-43 pathology whereas blocking this response exacerbates TDP-43 accumulation.

### HSF1(+) rescues TDP-43-induced cell death

**Figure 4:**
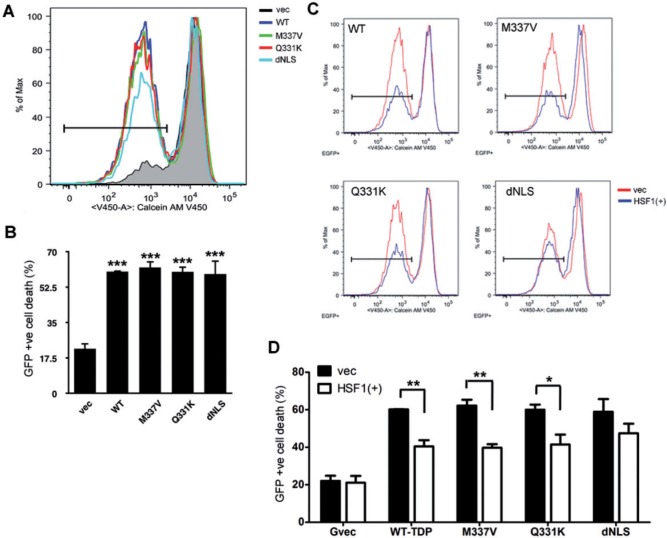
**Expression of HSF1(+) rescues TDP-43 toxicity.** ( **A** ) Cell death analysis performed on SH-SY5Y transfected with GFP-TDP-43 for 48 h. GFP-positive cells are plotted in this figure where dying cells are shown with reduced fluorescent intensity for calcine AM (as gated). GFP vector-expressing cells are indicated in shaded line whereas coloured lines represent the different genotypes of GFP-TDP-43 expressing cells. ( **B** ) Amount of dying GFP positive cells averaged from three independent transfections with SEMs displayed. Both wild-type and mutant TDP-43 are found to cause significant cell death compared to GFP vector control (one-way ANOVA followed by Bonferroni post-test, *P <* 0.001). ( **C** ) One representative experiment showing that the presence of HSF1(+) (blue line) reduces the level of dying GFP-TDP-43 expressing cells (red line). Average cell death from three transfections are shown in ( **D** ). *T* -test was used to determine whether HSF1(+) significantly rescues TDP-43 induced cell death. * *P <* 0.05; ^**^P < 0.01; ^***^P < 0.001.

As reported in other cellular and transgenic animal studies, overexpression of TDP-43 can be cytotoxic to some cells, especially neurons ( Ash *et al.* , 2010 ; Barmada *et al.* , 2010 ; Stallings *et al.* , 2010 ; Wils *et al.* , 2010 ). When either wild-type or ALS-associated mutant TDP-43 were overexpressed in the SH-SY5Y neuronal cell line, a significant reduction in cell survival was observed from those GFP-TDP-43 expressing cells ( Fig. 4 A and B and Supplementary Fig. 3 ), indicating that the overexpression of TDP-43 is indeed toxic to neuronal cells. This cytotoxicity can, however, be partially, but significantly rescued, by the presence of HSF1(+) ( Fig. 4 C and D). Similar findings were obtained in cells overexpressing ALS-linked mutant TDP-43. Conversely, although overexpression of GFP-dNLS TDP-43 resulted in similar levels of cell death as the other GFP-TDP-43 constructs, expression of HSF1(+) was only able to induce a non-significant ( *P =* 0.246) reduction in cell death. Overexpression of TDP-43 in HEK293 cells did not show cytotoxicity (data not shown), supporting the observation in ALS studies that neuronal cells are particularly sensitive to TDP-43 cytotoxicity.

In summary, we have demonstrated that overexpressing TDP-43 in cells recapitulates pathological features of the TDP-43 proteinopathies and that the presence of these features results in an increase in cell death. Manipulating the heat shock response (HSR) by expression of HSF1(+) reduces this pathological TDP-43, and improves cell survival. In contrast, when cells are deprived of their basal heat shock response activity by the expression of HSF1(−), the degree of TDP-43 proteinopathy is intensified.

### DNAJB2a as a potential mediator of HSF1-induced TDP-43 aggregation suppression

**Figure 5:**
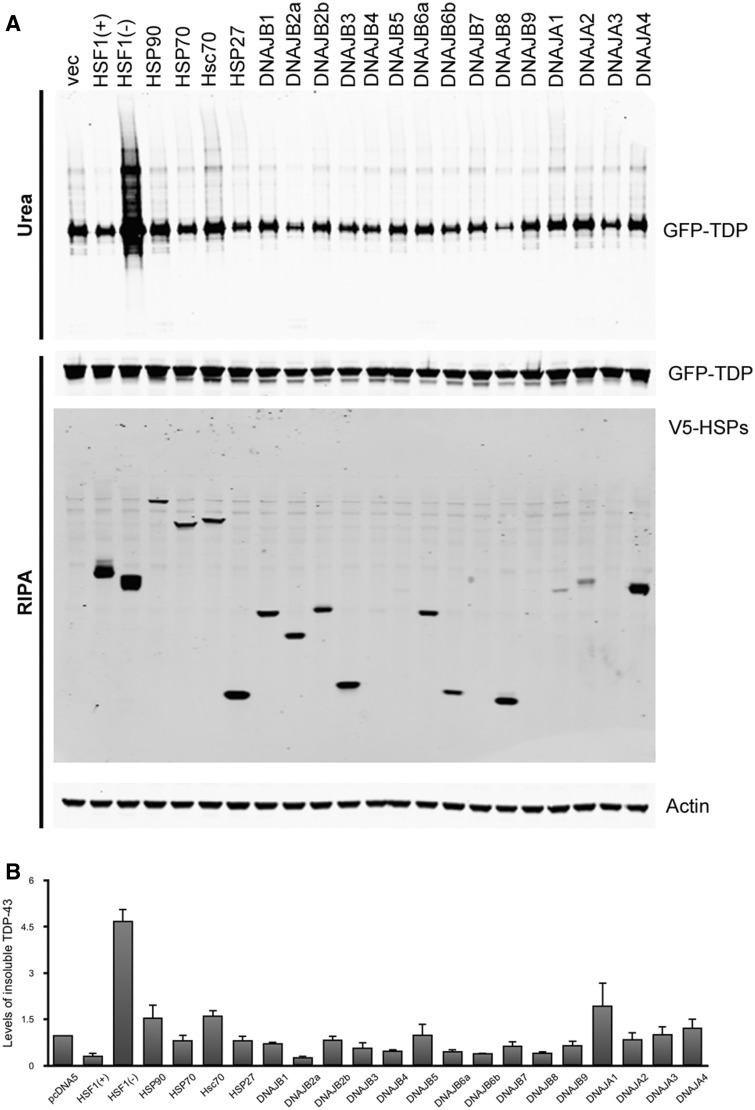
**Screening for HSPs that mediate HSF1(+)-induced TDP-43 clearance.** ( **A** ) HEK293T cells co-express GFP-TDP-43 and V5-HSP constructs for 48 h followed by fractionation. Levels of insoluble TDP-43 from three independent transfections are quantified, normalized to actin and shown in relative to control. Mean and SEM are shown in ( **B** ).

HSF1 is a transcription factor that is responsible for the induction of a wide range of HSPs and other stress-responsive proteins upon its activation ( Velichko *et al.* , 2013 ; Vihervaara *et al.* , 2013 ). As overexpression of HSF1(+) increased endogenous levels of HSP40 and HSP70, this indicated that HSF1(+) was triggering the heat shock response as expected ( Fig. 1 B). To determine which chaperone proteins may participate in TDP-43 clearance, we screened a selection of HSPs, including HSP90, HSP70, Hsc70, HSP27 and most of the HSP40s from the DNAJA and DNAJB families that were previously screened for their ability to suppress polyQ aggregation ( Hageman *et al.* , 2010 ). Most of the HSPs tested displayed little or no effect on TDP-43 solubility, however, two HSP40s, DNAJB2a and DNAJB8a, resulted in a reduction of insoluble TDP-43 similar to that observed with HSF1(+) ( Fig. 5 ). As DNAJB8a has a very restricted expression pattern and can only be found in testis tissue, we did not test this further. DNAJB2a displays a neuronal enriched expression profile ( Cheetham *et al.* , 1992 ; Chapple and Cheetham, 2003 ; Hageman and Kampinga, 2009 ), and was chosen for further investigation.

### DNAJB2a maintains the solubility of TDP-43 by interacting with HSP70

**Figure 6:**
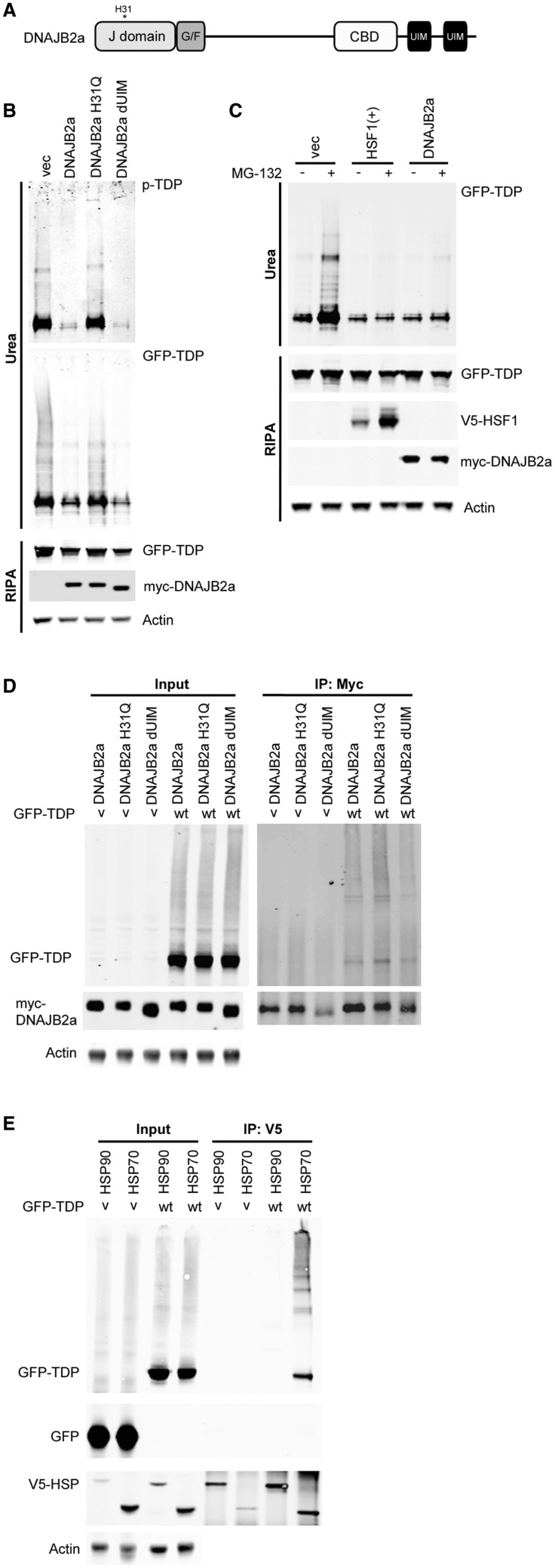
**DNAJB2a mediates TDP-43 refolding through introducing TDP-43 to HSP70.** ( **A** ) Domain structure of DNAJB2a, showing the position of the J (and H31), client binding (CBD) and UIM domains. ( **B** ) HEK293T cells co-expressing GFP-wild-type TDP-43 and myc-DNAJB2a constructs for 48 h followed by fractionation. Levels of insoluble TDP-43 are shown with GFP antibody and phospho-TDP-43 is detected by a phospho-TDP-43 specific antibody. ( **C** ) Cells co-expressing GFP-WT-TDP-43 and vector only (vec), HSF1(+), or myc-DNAJB2a were treated with the ubiquitin proteasome system inhibitor, MG-132 (0.5 μM, 40 h) followed by fractionation. ( **D** ) Co-immunopurification of GFP-TDP-43 and myc-DNAJB2a. Myc-DNAJB2a is pulled down by the myc antibody and GFP-TDP-43 is detected with TDP-43 antibody. ( **E** ) Co-immounopurification of GFP-TDP-43 and V5-HSFP70. V5-HSP70 and V5-HSP90 are pulled down by V5 antibody and GFP-TDP-43 is detected with the GFP antibody.

DNAJB2a, encoded by *DNAJB2* and also known as HSJ1a, is a target of HSF1 ( Vihervaara *et al.* , 2013 ) and functions as a co-chaperone for HSP70 interacting through its J domain ( Fig. 6A; Cheetham *et al.* , 1994 ). In addition to working with HSP70, DNAJB2a also binds client proteins independently and can target them to the proteasome for degradation through its ubiquitin interacting motif (UIM) ( Fig. 6A; Westhoff *et al.* , 2005 ). To investigate the nature of this HSF1-DNAJB2a-mediated TDP-43 aggregation clearance, we expressed GFP-wild-type TDP-43 with either wild-type or selected mutants of DNAJB2a. The H31Q mutation in DNAJB2a disrupts the interaction between DNAJB2a and HSP70, thereby impeding the co-chaperone activity of DNAJB2a. The DNAJB2a-dUIM bears four point mutations in the UIM, which prevent DNAJB2a from delivering client proteins to the proteasome for degradation ( Westhoff *et al.* , 2005 ). Interestingly, the DNAJB2a-mediated reduction in insoluble/phosphorylated TDP-43 was not altered by the presence of the dUIM mutations, whereas the presence of the H31Q mutation completely disrupted this activity ( Fig. 6 B). These findings suggest that DNAJB2a reduces the level of insoluble TDP-43 by co-operating with HSP70 to enhance refolding and maintain solubility, rather than by causing the degradation of the insoluble protein. In support of this hypothesis, treatment of GFP-WT-TDP-43 expressing cells with the proteasome inhibitor, MG-132, which led to the build-up of insoluble TDP-43 ( Fig. 6 C), had minimal impact on HSF1(+)/DNAJB2a-mediated TDP-43 aggregation suppression ( Fig. 6 C). Both the DNAJB2a functional mutations study and the study of ubiquitin proteasome system inhibition by MG-132 suggest that protein degradation does not play a major role in HSF1-DNAJB2a-mediated TDP-43 aggregation clearance. This is further supported by our observation that autophagy inhibition by bafilomycin treatment showed minimal effect on HSF1(+)/DNAJB2a-mediated TDP-43 aggregation clearance ( Supplementary Fig. 4 ).

An immunopurification study provided further evidence to support the existence of a DNAJB2a-HSP70-TDP-43 refolding complex, since TDP-43 was shown to interact with both DNAJB2a and HSP70 ( Fig. 6 D and E). Although the co-chaperone’s binding to the client protein is in general transient and dynamic, we were able to detect TDP-43 binding to all three forms of DNAJB2a. The binding to H31Q-DNAJB2a was stronger than the others, probably due to its inability to transfer the client protein to HSP70, therefore prolonging binding ( Fig. 6 D).

These results show that the HSF1-induced co-chaperone, DNAJB2a, reduced levels of insoluble TDP-43 not through degrading these aggregated, phosphorylated proteins, but by directing them to HSP70 to be refolded ( Fig. 6 and Supplementary Fig. 5 ). As TDP-43 is tightly controlled to maintain its cellular level, and is known to be aggregation-prone especially under stress, our finding suggests a promising therapeutic strategy which uses the heat shock response mechanism to regain the neutral TDP-43 protein homeostasis.

### HSF1/heat shock proteins are reduced in ALS mouse model and affected patient tissues

The HSR is a powerful mechanism to cope with cellular stress, which we show in this study can efficiently refold insoluble TDP-43 and dissolve features of TDP-43 proteinopathy; however, it has been shown to be compromised in some neurodegenerative conditions ( Hay *et al.* , 2004 ; Maheshwari *et al.* , 2014 ). To characterize further the involvement of HSF1/HSPs in TDP-43 proteinopathy in ALS, levels of HSF1 and HSPs proteins were investigated in ALS mouse model and patient tissues.

To study the role of TDP-43 in the development and progression of ALS, lines of transgenic mice expressing either wild-type-TDP-43, Q331K-TDP-43 or both were established ( Mitchell *et al.* , 2015 ). A correlation between the level of TDP-43 protein and phenotypic severity was noted, with the strain that expressed the highest level of TDP-43 (TDP-43 ^WTxQ331K^ double transgene) suffering from the most severe motor deficits and TDP-43 pathology, and a drastically reduced life span compared to both single and non-transgenic mice ( Mitchell *et al.* , 2015 ).

**Figure 7:**
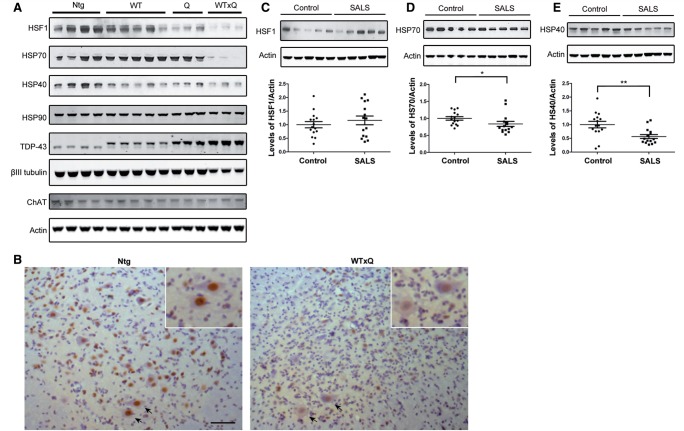
**The HSR is downregulated in the affected tissues of both TDP-43 mouse model and sporadic ALS.** ( **A** ) Whole spinal cord lysates are prepared from 8-week-old non-transgenic (Ntg), wild-type TDP-43 single transgenic (WT), Q331K-TDP-43 single transgenic (Q) and WTxQ331K-TDP-43 double transgenic (WTxQ) mice. Endogenous and transgenic TDP-43 protein levels are shown with TDP-43 blotting; components of the HSR are shown with HSF1, HSP70, HSP40 and HSP90; neuronal markers, βIII tubulin and ChAT, are also shown for indication of neuronal loss. Actin is used as a loading control. ( **B** ) Immunohistochemistry staining for HSF1 in spinal cord sections of non-transgenic and double transgenic animals at 8 weeks old. Selective motor neurons indicated by arrows, and inset. Scale bar = 50 μm. Levels of HSF1 ( **C** ), HSP70 ( **D** ), and HSP40 ( **E** ) of control and sporadic ALS spinal cord lysate are shown. 15 control and 15 sporadic ALS (SALS) samples were used in this study, representative western blots are shown with all samples plotted for *t* -test comparison. * *P <* 0.05; ^**^P < 0.01.

Interestingly, HSF1 was decreased in the TDP-43 ^Q331K^ single transgenic mice that displayed a mild, non-lethal motor defect at later stage ( Fig. 7 A and Mitchell *et al.* , 2015 ), but was most significantly lost in diseased TDP-43 ^WTxQ331K^ mice ( Fig. 7 A). As a result of the loss of HSF1, levels of HSP70 and HSP40 were also decreased substantially in TDP-43 ^WTxQ331K^ mice, whereas HSP90 remained unaffected. Immunohistochemical staining also showed reduced levels of HSF1 in the remaining motor neurons in the end-stage diseased mice spinal cord sections, ( Fig. 7 B) indicating that the reduction in HSF1 protein was not purely a result of motor neuron loss.

Examination of HSF1 and the HSPs in human tissue identified a significant decrease in HSP70 ( Fig. 7 D) and HSP40 ( Fig. 7 E) despite no significant change in HSF1 levels between sporadic ALS and control tissues ( Fig. 7 C). It is known that total level of HSF1 is not always correlated with its level of activity, instead, certain post-translational modifications are required to activate the protein ( Westerheide *et al.* , 2009 ; Calderwood *et al.* , 2010 ; Yih *et al.* , 2012 ). This regulation in HSF1 activity is likely to be responsible for the difference we see in affected tissues in ALS, where HSP40 and 70 are reduced, despite no apparent change in the total levels of HSF1. We therefore propose that a reduction in HSF1 activity results in a reduction in HSPs, which in turn contributes to the enhanced accumulation and aggregation of TDP-43 in ALS patients.

Taken together, our results suggest that the HSR is compromised in both the TDP-43 transgenic ALS mouse model and in patient tissues, which could be initiated either directly or indirectly by long term TDP-43 protein overexpression. Additionally, these results demonstrate that a reduction in the HSR leads to decreased TDP-43 refolding efficiency, promoting the accumulation of phosphorylated insoluble TDP-43.

## Discussion

**Figure 8:**
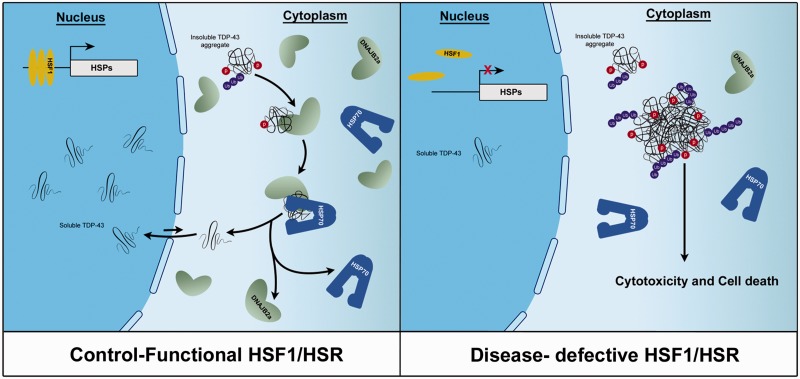
**Proposed model of HSR involvement in TDP-43 protein refolding and proteinopathy.** In the healthy condition with a functional HSR, HSF1 activates the expression of HSPs such as HSP70 and DNAJB2a. DNAJB2a recognizes misfolded TDP-43 in the cytosol and brings it to the chaperone protein, HSP70. HSP70 catalyzes the refolding of TDP-43, returns it to its native soluble state, which enables TDP-43 to shuttle back to the nucleus. In the diseased condition, the HSR is impaired by either reduced protein levels or the decreased activity of HSF1. In either condition, this leads to a reduction of HSPs, which subsequently fail to efficiently engage misfolded TDP-43. As a result, the insoluble and phosphorylated TDP-43 proteins accumulate and form cytosolic aggregates in disease affected cells.

In this study, we generated a cellular model of the human TDP-43 proteinopathies that developed detergent-resistant, hyperphosphorylated cytoplasmic insoluble TDP-43 aggregates that are cytotoxic. Co-expression of a dominant positive version of the master regulator of the heat shock response HSF1 induced a dramatic clearance of insoluble TDP-43 aggregates without affecting the levels of soluble TDP-43. Conversely the dominant negative version of HSF1 lead to a massive accumulation of insoluble and hyperphosphorylated TDP-43 aggregates confirming a vital role of the HSF1-HSP axis in physiological TDP-43 proteostasis. We have shown that HSF1-induced TDP-43 clearance is partly mediated by HSP70 and its co-chaperone DNAJB2a in a proteasome- and autophagy-independent manner. We propose that DNAJB2a recognizes and binds to insoluble TDP-43 taking it to the chaperone protein HSP70, which then refolds the protein. Once it has been returned to its soluble, properly folded status, TDP-43 is then released to perform its biological functions ( Fig. 8 ). Because DNAJB2a mediates TDP-43 refolding without involving the proteasome or autophagy, it is able to reduce TDP-43 toxicity without changing the levels of soluble TDP-43.

We have also shown that levels of several HSR proteins are reduced in transgenic mice and sporadic ALS tissues implying that the HSF1–HSP axis may be compromised due to the disease process. Q331K TDP-43 transgenic mice develop a slowly progressive motor phenotype and have moderately reduced levels of HSF1 and HSP-40, whereas the severely-affected double transgenic mice have a dramatic loss of HSF1, HSP40 and HSP70. In human spinal cord tissues the levels of HSP70 and HSP40 were significantly reduced but not HSF1.

The data from our cellular, animal and human tissue studies indicate that the HSF1/HSP70/DNAJB2a pathway plays a vital role in the physiological maintenance of TDP-43 proteostasis. In the disease state, however, the HSR appears to be compromised and may contribute to the accumulation of insoluble TDP-43 protein aggregates and exacerbate defective proteostasis ( Fig. 8 ).

### Involvement of heat shock proteins in neurodegenerative disorders

Molecular chaperones are essential for maintaining proteostasis ( Smith *et al.* , 2015 ). Neurodegenerative disorders are characterized by the presence of aggregated aberrant proteins in affected neural tissues, so the role of HSR proteins and the opportunities for therapy is of great interest. Reduced levels of HSR components are found in affected neuronal tissues from patients with Huntington’s disease ( Maheshwari *et al.* , 2014 ) and ALS ( Anagnostou *et al.* , 2010 ) and a failed stress response has been implicated in ageing and neurodegenerative disorders ( Brehme *et al.* , 2014 ). Overexpression of individual HSPs in cellular and transgenic animal models of disease have shown variable degrees of success. In some cases, overexpression of a single HSP was sufficient to reduce protein aggregation and improve survival, such as: HSP70 for α-synuclein ( Auluck *et al.* , 2002 ; Dedmon *et al.* , 2005 ), DNAJB6b and DNAJB8 for poly-Q ( Hageman *et al.* , 2010 ), HSP27 for tau and SOD1 ( Shimura *et al.* , 2004 ; Sharp *et al.* , 2008 ) and HSPB8 for SOD1 ( Crippa *et al.* , 2010 ). In other cases, both HSP70 and HSP40 were required to clear aberrant protein aggregates ( Krobitsch and Lindquist, 2000 ; Lotz *et al.* , 2010 ). The differences are likely to be due to substrate specificity and the particular model used in the study. The HEK293T cells used in our study have high levels of HSP70 in basal conditions but relatively low levels of HSP40 ( Fig. 1 B). As the level of HSP40 was a limiting factor in our cellular model, it may explain why we observed no rescue of TDP-43 pathology in response to HSP70 overexpression alone ( Fig. 5 A), despite evidence that the interaction between HSP70 and DNAJB2a is essential for insoluble TDP-43 clearance ( Fig. 6 B). Unlike HSP70, DNAJB2a expression is specifically enriched in neuronal tissues ( Chapple and Cheetham, 2003 ; Hageman and Kampinga, 2009 ) and mutations in DNAJB2 are linked to a rare recessive distal hereditary motor neuropathy and Charcot–Marie–Tooth disease type 2 ( Blumen *et al.* , 2012 ; Gess *et al.* , 2014 ) implying a critical need for this co-chaperone. Overexpression of DNAJB2a was protective in G93A-SOD1 mouse model of ALS ( Novoselov *et al.* , 2013 ) and mutant huntingtin in R6/2 transgenic mice ( Labbadia *et al.* , 2012 ). These results, in combination with our own data on TDP-43, provide convincing evidence that DNAJB2a plays an important role in neuronal survival and the capacity of neurons to cope with aberrant protein aggregation. Interestingly, both the refolding (J) and proteasome (UIM) domains of DNAJB2a were required for mutant SOD1 and huntingtin protein clearance, whereas we demonstrated that TDP-43 clearance was achieved by HSP70/DNAJB2a-mediated refolding and not proteasome clearance. The marked reduction in TDP-43 C-terminal phosphorylation indicates that the protein is in a near native and probably functional state. A similar result was seen where DNAJB2a suppressed the aggregation of mutant parkin and enhanced mitophagy in a J domain-dependent and UIM-independent manner ( Rose *et al.* , 2011 ). This result demonstrates that the diverse function of DNAJB2a to mediate protein clearance either through refolding or degradation depends on the nature and the structure of the protein client.

Interactions between HSPs and TDP-43 have been documented previously. Freibaum *et al.* (2010) conducted a proteomic screening which identified a group of HSPs interacting with TDP-43, including HSP70 and HSP40. These findings were later validated in cellular model ( Chang *et al.* , 2013 ; Udan-Johns *et al.* , 2014 ). Both studies suggested that the HSP-TDP-43 interaction plays a role in stress-induced TDP-43 aggregation. However, as the HSP expression pattern is cell type specific, the exact role of the HSP-TDP-43 interaction in the context of neurodegeneration is still yet fully explored.

### Cellular stress and TDP-43 aggregation: is heat shock response activation enough to help?

TDP-43 is an aggregation-prone protein that is found to form detergent-resistant aggregates or stress granules during conditions of cellular stress such as heat shock ( Supplementary Fig. 6 ; Udan-Johns *et al.* , 2014 ), osmotic, and oxidative stress ( Dewey *et al.* , 2011 ; McDonald *et al.* , 2011 ; Chang *et al.* , 2013 ). Studies in cellular models show that despite TDP-43 aggregates forming quickly in response to stress, those aggregates can be readily disassembled when the stress is removed ( Supplementary Fig. 6 ; Scotter *et al.* , 2014 ). Several cellular mechanisms have evolved to participate in resolving potentially detrimental protein aggregates. The ubiquitin proteasome system has been found to be responsible for the majority of TDP-43 protein degradation, whereas the autophagy pathway is required to clear larger aggregates ( Scotter *et al.* , 2014 ). Indeed, we observed an increase in TDP-43 aggregation when the proteasome was inhibited by MG132 ( Fig. 6 B); however, neither proteasome nor autophagy inhibition reduced the effectiveness of HSF1 or DNAJB2a overexpression, further confirming that they do not appear to be targeting TDP-43 for degradation. Upstream of these pathways, the chaperone system facilitates protein folding and directs the degradation of misfolded proteins. However, due to their highly specified structures, neurons have higher demand for these chaperone and protein degradation systems to maintain protein homeostasis, which appear to fail in ageing and disease ( Brehme *et al.* , 2014 ). In addition, motor neurons have a higher threshold for the activation of the HSR than most other cells ( Batulan *et al.* , 2003 ). Thus there may be a delay in the activation of the chaperone system in response to stress, creating a cascade where the protein quality and degradation systems begin to fail and aberrant protein aggregates form. If and when, the HSR eventually does come into play, it may be too little too late. Indeed, there is no evidence to date to suggest that the HSR is activated in diseased tissues and may actually be inhibited ( Fig. 7 ) ( Anagnostou *et al.* , 2010 ; Maheshwari *et al.* , 2014 ). Our data suggest that the enforced activation of the HSR, in particular the neuronal specific co-chaperone, DNAJB2a, could effectively rescue TDP-43 proteinopathy by restoring aggregated TDP-43 to its native state.

HSP90 binds to and inactivates HSF1 by preventing it from entering the nucleus and inducing the HSR. HSP90 inhibitors such as 17-AAG have therefore been tested in cellular and animal models of neurodegeneration with some success ( Waza *et al.* , 2005 ; Fujikake *et al.* , 2008 ; Rusmini *et al.* , 2011 ; Gregory *et al.* , 2012 ). However, as HSP90 is involved in cell cycle and cell survival pathways ( Taipale *et al.* , 2010 ), the risk of toxic side effects of HSP90 inhibitors limit their attractiveness as a potential therapy. We have shown that activation of HSF1/HSP70/DNAJB2a pathway results in a dramatic clearance of TDP-43 aggregates and that directly targeting these HSR components is an important therapeutic opportunity for ALS.

## Supplementary Material

Supplementary Data

## Abbreviations

ALS: amyotrophic lateral sclerosis;
GFP: green fluorescent protein;
HSP: heat shock protein;
HSR: heat shock response;
UIM: ubiquitin interacting motif
