# Supplementary material for: The heat shock response plays an important role in TDP-43 clearance: evidence for dysfunction in amyotrophic lateral sclerosis
Source: Brain. 2016 Mar 1;139(5):1417–32. doi: 10.1093/brain/aww028 (PMC4845254; doi:10.1093/brain/aww028)
Supplement: Supplementary Data [file aww028_supplementary_data.zip › brain-2015-01677-File002.pdf]

Supplementary materials

The heat shock response plays an important role in TDP-43 clearance: evidence for dysfunction in Amyotrophic Lateral Sclerosis

Han-Jou Chen[1], Jacqueline C Mitchell[1], Sergey Novoselov[2], Jack Miller[1], Agnes L Nishimura[1], Emma L Scotter[3], Caroline A Vance[1], Michael E Cheetham[2], Christopher E Shaw[1*]

Supplementary Material and Methods

NSC-34 cells (a gift from Dr. Neil Cashman) and GFP-WT TDP-43 inducible HEK cells were cultured using DMEM and DMEM/F12 (Life Technologies) supplemented with 10% FBS (Life Technologies), and maintained at 37°C, 5% CO^{2}. Cells were plated a day before the experiment and media was refreshed prior to plasmid DNA transfection using Lipofectamine 2000 (Life Technologies). 1 μg/ml of doxycycline was used for induction.

Supplementary Figure 1

Solubility and subcellular distribution of endogenous TDP-43. (A, B) Levels of endogenous TDP-43 in soluble and insoluble fractions from 2 day HSF1 transfected HEK cells are shown (A) and quantified (B). HSF1 had no significant effect on endogenous TDP-43 protein levels in either fraction (one-way ANOVA, $p>0.5$). **(C)** Endogenous TDP-43 was assessed using an antibody specifically recognizing mouse TDP-43 in GFP-TDP-43 one day transfected NSC-34 cells. Endogenous TDP-43 was found to localize to nuclei in untransfected cells, However it was found to co-localise with exogenous TDP-43 in both large (arrowhead) or small (arrows) cytosolic aggregates

Supplementary Figure 2

Subcellular distribution of GFP-TDP-43 co-expressed with HSF1. Distribution of GFP-TDP-43 was quantified from images of HEK cells co-expressed GFP TDP-43 and HSF1. Five confocal images are taken from each condition each experiment and analyzed with the imaging analysis software MetaMorph. Three independent experiments were done in total. Mean and SEM are shown.

Supplementary Figure 3

TDP-43 cytotoxicity in SH-SY5Y cells. Cell death analysis performed on SH-SY5Y cells transfected with GFP-TDP-43 for 48 hours. Cells were stained with APC-

annexin V and analysed by BD FACSCalibur. The proportion of annexin V-positive cells in GFP expressing cells is shown. Both WT and mutant TDP-43 are found to cause significant cell death compared to GFP vector control (one-way ANOVA $p<0.001$, followed by Bonferroni post-test* $p<0.05$; ** $p<0.01$; *** $p<0.001$; **** $p<0.0001$).

Supplementary Figure 4

Autophagy inhibition on HSF1(+)- or DNAJB2a-mediated TDP-43 refolding. HEK 293T cells co-expressing GFP-WT TDP-43 and vector only, V5-HSF1(+) or myc-DNAJB2a were treated with the autophagy inhibitor, bafilomycin (100 nM, 24 hours) followed by fractionation. LC3 and p62 were shown as markers for autophagy inhibition (n=3).

Supplementary Figure 5

HSF1(+) and DNAJB2a refold already existing TDP-43 aggregates. (A) Experimental plan for this study where GFP-TDP-43 was induced by the addition of doxycycline for 48 hours. GFP-TDP-43 expression was then turned off when HSF1 or DNAJB2a was transfected for another 48 hours. **(B)** HSF1(+) and DNAJB2a were found to reduce the level of insoluble GFP-TDP-43 which accumulated in the first 48 hour period (n=3).

Supplementary Figure 6

Dynamic of TDP-43 protein solubility in response to heat shock. HEK293T cells expressing GFP-WT TDP-43 were heat shocked at 42℃ for indicated period of time. Cells were harvested and fractionated at the indicated time point after heat shock or 120 minutes of heat shock with recovery at 37℃ overnight (120+RC).
