# Supplementary figures and images for: The heat shock response plays an important role in TDP-43 clearance: evidence for dysfunction in amyotrophic lateral sclerosis
Source: Brain. 2016 Mar 1;139(5):1417–32. doi: 10.1093/brain/aww028 (PMC4845254; doi:10.1093/brain/aww028)

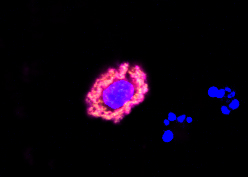

Supplement: Supplementary Data [file aww028_supplementary_data.zip › brain-2015-01677-File017.jpg]

A.

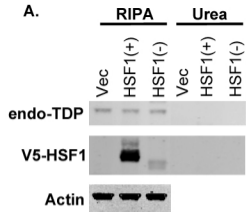

B.

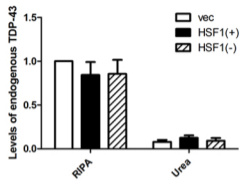

C.

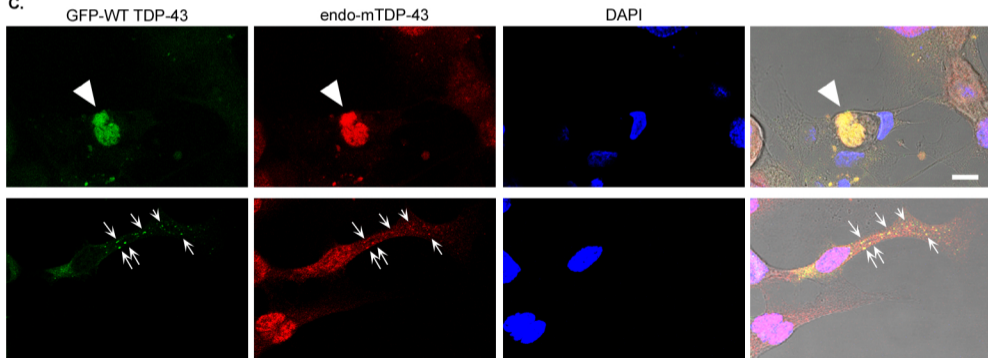

Supplement: Supplementary Data [file aww028_supplementary_data.zip › brain-2015-01677-File011.pdf]

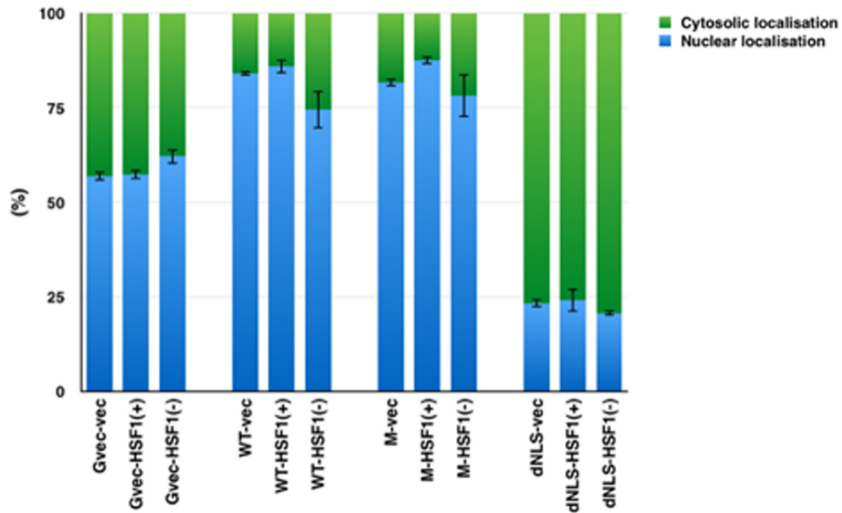

Supplement: Supplementary Data [file aww028_supplementary_data.zip › brain-2015-01677-File012.pdf]

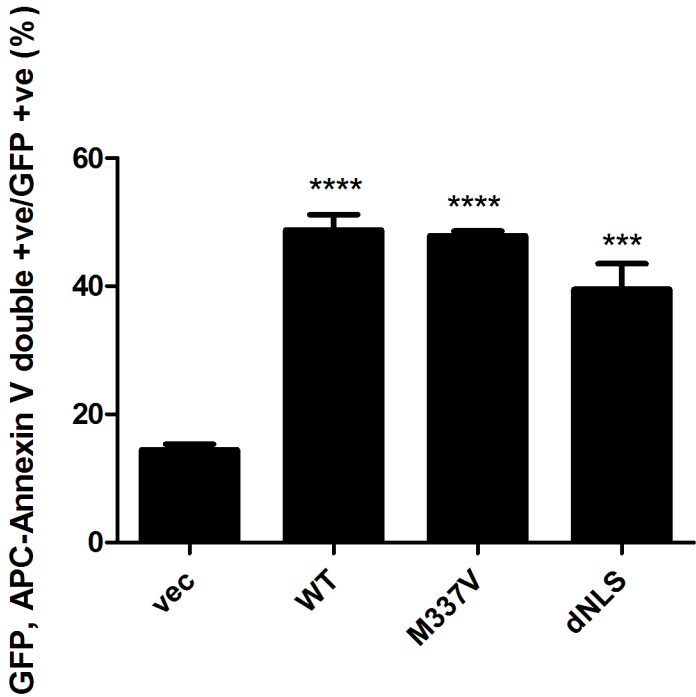

Supplement: Supplementary Data [file aww028_supplementary_data.zip › brain-2015-01677-File013.pdf]

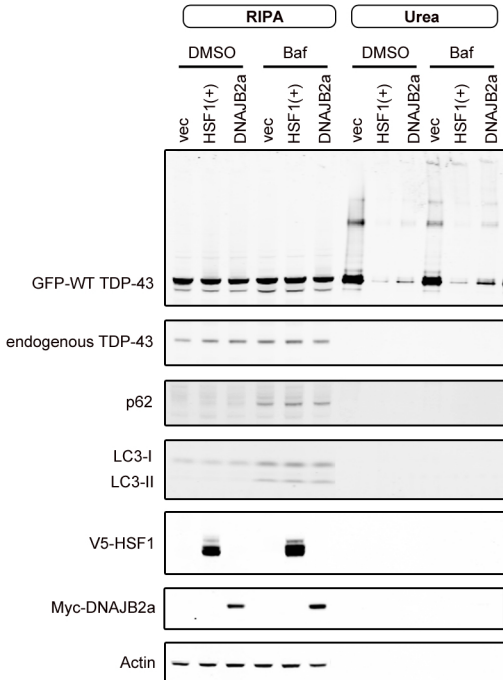

Supplement: Supplementary Data [file aww028_supplementary_data.zip › brain-2015-01677-File014.pdf]

**A.**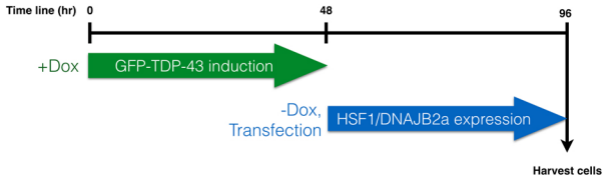**B.**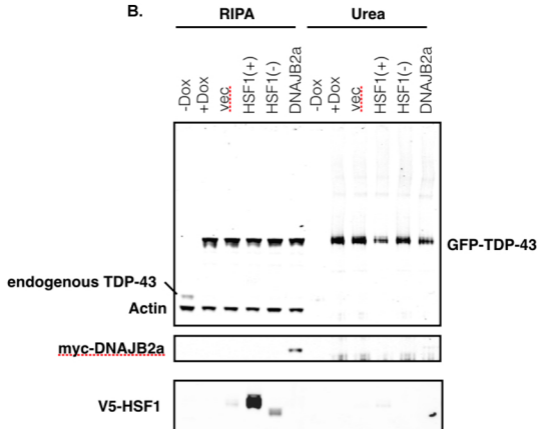

Supplement: Supplementary Data [file aww028_supplementary_data.zip › brain-2015-01677-File015.pdf]

Heat Shock (min)

GFP-TDP-43

GFP-TDP-43

Actin

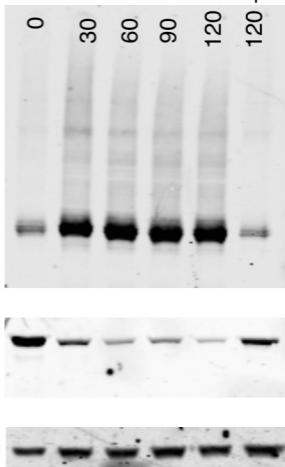

Urea

RIPA

Supplement: Supplementary Data [file aww028_supplementary_data.zip › brain-2015-01677-File016.pdf]
